# IRONMAN: A Novel International Registry of Men With Advanced Prostate Cancer

**DOI:** 10.1200/GO.22.00154

**Published:** 2022-11-04

**Authors:** Lorelei A. Mucci, Jacob Vinson, Theresa Gold, Travis Gerke, Julie Filipenko, Rebecca M. Green, Simon G. Anderson, Simone Badal, Anders Bjartell, Kim N. Chi, Ian D. Davis, Deborah Enting, André P. Fay, John Lazarus, Joaquin Mateo, Ray McDermott, Folakemi T. Odedina, David Olmos, Aurelius Omlin, Ademola A. Popoola, Camille Ragin, Robin Roberts, Kjell M. Russnes, Charles Waihenya, Konrad H. Stopsack, Terry Hyslop, Paul Villanti, Philip W. Kantoff, Daniel J. George

**Affiliations:** ^1^ Harvard T.H. Chan School of Public Health, Boston, MA; ^2^ Prostate Cancer Clinical Trials Consortium, New York, NY; ^3^ The Glasgow-Caribbean Centre for Development Research and the Caribbean Institute of Health Research, The University of the West Indies, Bridgetown, Barbados; ^4^ The University of the West Indies Mona, Kingston, Jamaica; ^5^ Skåne University Hospital, Malmö, Sweden; ^6^ BC Cancer, Vancouver Prostate Centre, University of British Columbia, Vancouver, British Columbia, Canada; ^7^ Monash University, Melbourne, Australia; ^8^ Eastern Health, Melbourne, Australia; ^9^ Guys St Thomas NHS Foundation Trust, London, United Kingdom; ^10^ Hospital Sao Lucas da PUCRS, Porto Alegre, Brazil; ^11^ University of Cape Town, Cape Town, South Africa; ^12^ Vall d’Hebron Institute of Oncology, Barcelona, Spain; ^13^ St Vincent's University Hospital & Cancer Trials Ireland, Dublin, Ireland; ^14^ Mayo Clinic, Jacksonville, FL; ^15^ Hospital Universitario 12 de Octubre, Instituto de Investigación Sanitaria Hospital 12 de Octubre (imas12), Madrid, Spain; ^16^ Kantonsspital St Gallen, St Gallen, Switzerland; ^17^ University of Ilorin, Ilorin, Nigeria; ^18^ Fox Chase Cancer Center, Philadelphia, PA; ^19^ UWI School of Clinical Medicine and Research, Nassau, The Bahamas; ^20^ Oslo University Hospital, Oslo, Norway; ^21^ University of Nairobi, Nairobi, Kenya; ^22^ Duke Cancer Institute, Durham, NC; ^23^ Movember Foundation, Melbourne, Australia; ^24^ Convergent Therapeutics, Cambridge, MA; ^25^ Memorial Sloan Kettering Cancer Center, New York, NY

## Abstract

To describe a newly established international registry recruiting diverse patients with advanced prostate cancer across academic and community practices to address unmet needs in this population.

**PATIENTS AND METHODS:**

Initiated in 2017, IRONMAN (International Registry for Men with Advanced Prostate Cancer) is a prospective cohort of patients with advanced prostate cancer. The study will enroll 5,000 patients with metastatic hormone-sensitive prostate cancer (mHSPC) or castration-resistant prostate cancer (CRPC), recruited from Australia, the Bahamas, Barbados, Brazil, Canada, Ireland, Jamaica, Kenya, Nigeria, Norway, South Africa, Spain, Sweden, Switzerland, the United Kingdom, and the United States. The study is collecting datatypes to study variation in care and treatment of advanced prostate cancer across countries and across academic, community-based, and government practices with a focus on clinical outcomes, patient-reported outcomes, epidemiologic data, biologic subtypes, and clinician questionnaires.

**RESULTS:**

Through July 2022, 2,682 eligible patients were enrolled in 11 of 12 active countries. Sixty-six percent of patients have mHSPC, and 34% have CRPC. On the basis of self-report, 11% of patients are Black and 9% are Hispanic. Five Veterans Affairs Medical Centers are enrolling patients. Globally, 23% of patients report being veterans of military service.

**CONCLUSION:**

To our knowledge, this is the first international cohort of people newly diagnosed with advanced prostate cancer designed to describe variations in patient management, experiences, and outcomes. IRONMAN aims to identify optimal treatment sequences to improve survival, understand patient-reported outcomes, and explore novel biomarkers to understand treatment resistance mechanisms. Insights from IRONMAN will inform and guide future clinical management of people with mHSPC and CRPC. This cohort study will provide real-world evidence to facilitate a better understanding of the survivorship of people with advanced prostate cancer.

## INTRODUCTION

Globally, 1.4 million people are diagnosed annually with prostate cancer and 487,000 die of the disease.^1^ In the United States, 3.6 million people are prostate cancer survivors,^2^ of whom an estimated 180,000 are living with advanced prostate cancer, defined as de novo metastatic hormone-sensitive prostate cancer (mHSPC) at diagnosis; cancers that have progressed to mHSPC after local therapy, and cancers that have progressed to castration-resistant prostate cancer (CRPC). Given the lack of population-level data on patients with advanced prostate cancer, this number is an estimate and the exact number globally is unknown.

**CONTEXT**

**Key Objective**
In the United States, 180,000 patients are advanced prostate cancer survivors. The clinical landscape for these patients has rapidly evolved over the past decade, yet relatively little is known about the real-world patient experience of this group of patients with advanced cancer. The key objective of IRONMAN is to collect diverse data elements in a prospective and international setting to provide information on the survivorship experience and unmet needs of patients with advanced prostate cancer.
**Knowledge Generated**
Our findings support our ability to successfully enroll an international cohort of patients with newly diagnosed advanced prostate cancer, to collect high-quality and diverse data elements, and to engage a diverse cohort on the basis of race, age, geography, and veterans of military service.
**Relevance**
Real-world data, such as those generated by IRONMAN, can inform the potential optimal sequences of therapeutic agents, identify subgroups of patients who would benefit, and inform future clinical trials.

The survivorship needs for advanced prostate cancer are distinct from localized disease. These patients are at greatest risk of cancer death^3^ and experience poorer quality of life because of the severity of disease and its therapies. The treatment landscape has changed considerably over the past decade with 11 new therapies approved in the United States, each of which is associated with a modest survival advantage (Appendix Table A1).^4-15^ There is variability globally in access to these newer therapies, whereas some patients are using older lines of therapies. There are a lack of clear consensus on the optimal treatment patterns in the advanced disease setting and likely variability in treatment patterns across practices.^15^ There is a paucity of data on real-world quality of life, risk of adverse events or comorbidities, and the potential impact of lifestyle factors on outcomes for patients with advanced prostate cancer.

**FIG 1:**
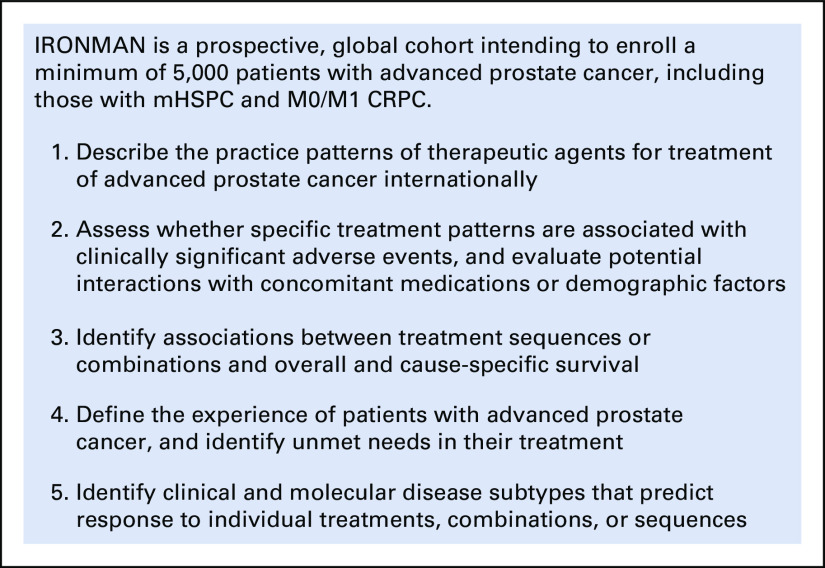
Objectives of the IRONMAN registry. CRPC, castration-resistant prostate cancer; mHSPC, metastatic hormone-sensitive prostate cancer.

**FIG 2:**
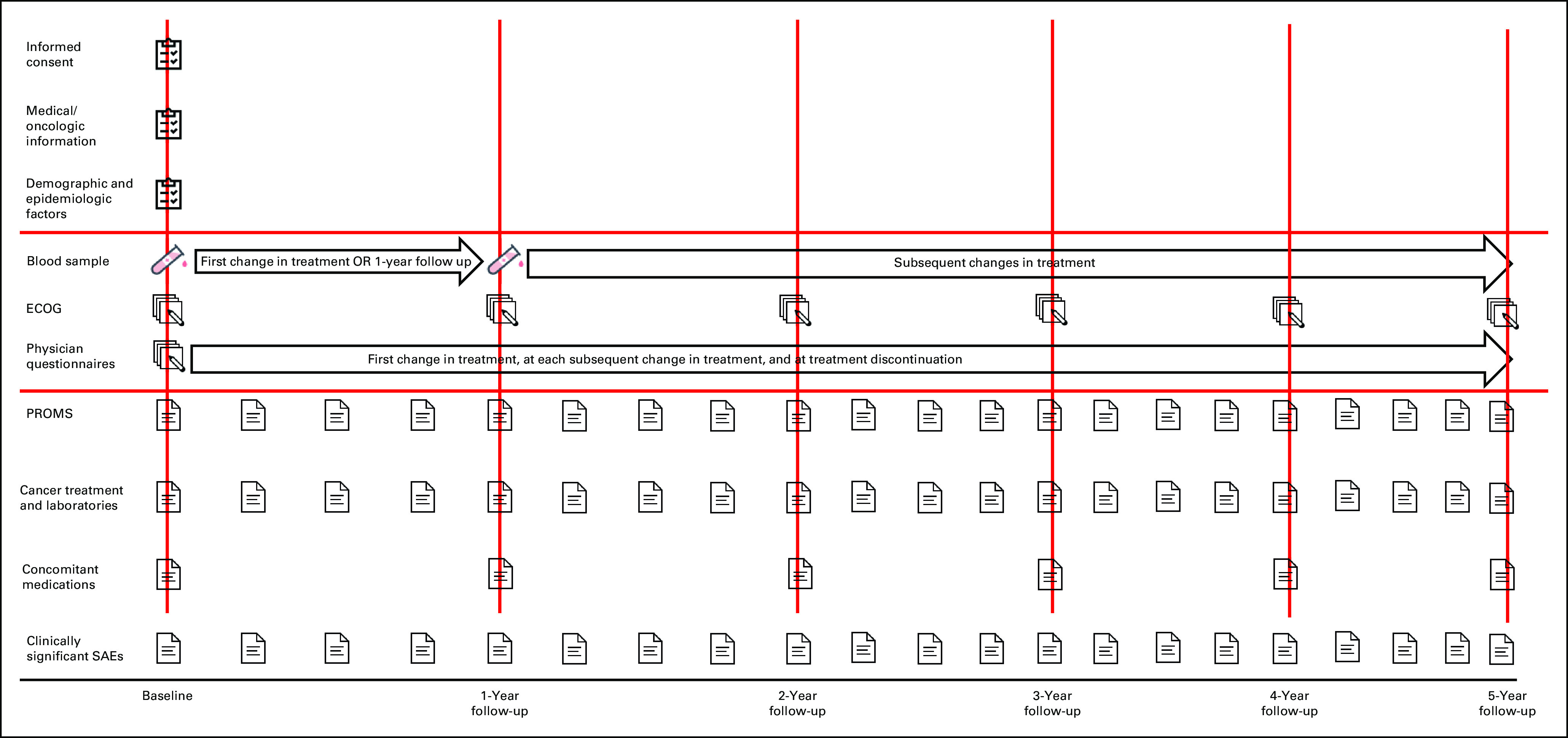
Overview of the IRONMAN study design.

**FIG 3:**
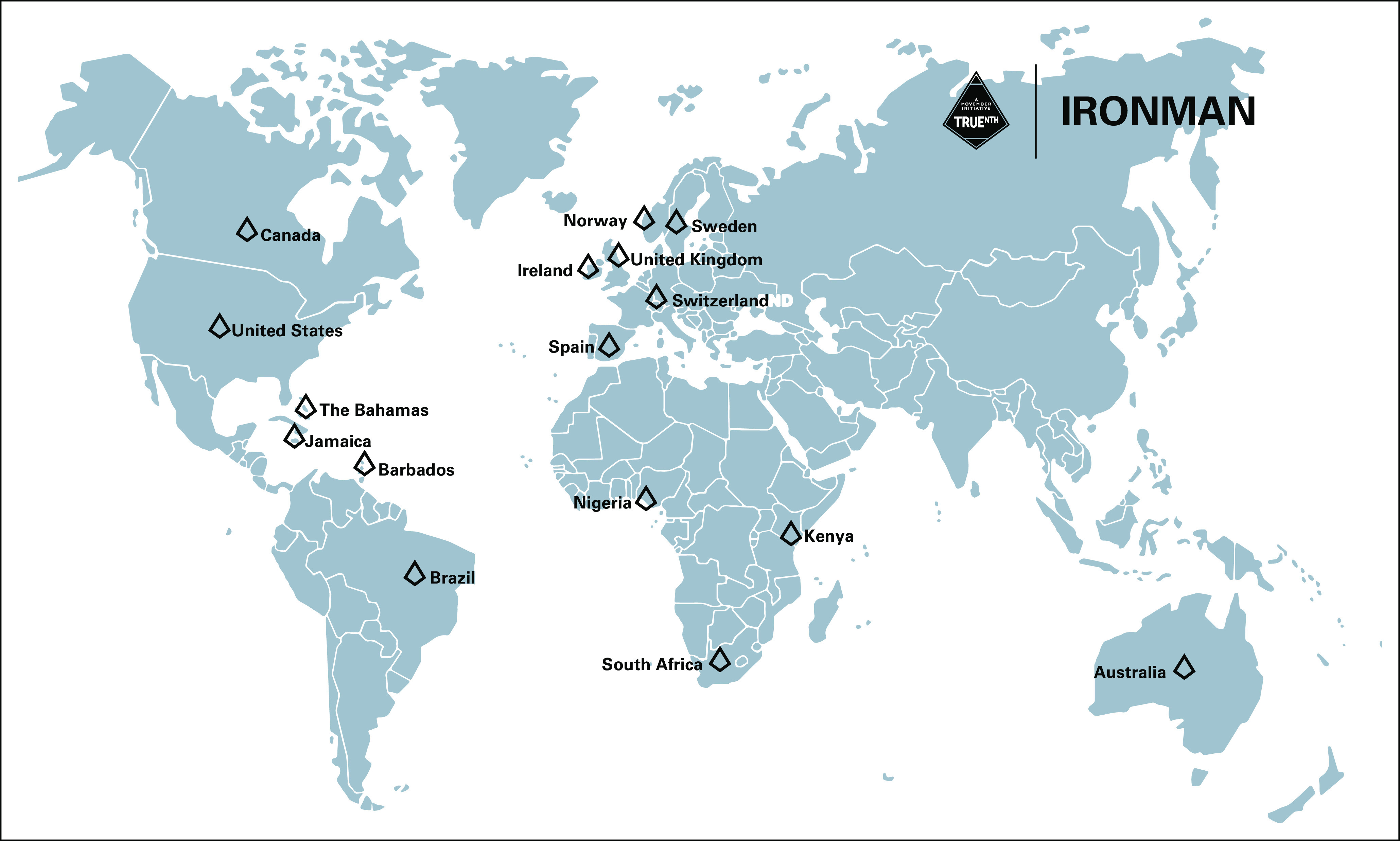
Countries with participating IRONMAN sites.

IRONMAN (International Registry for Men with Advanced Prostate Cancer; ClinicalTrials.gov identifier: NCT03151629) was designed to address the outstanding issues for people with advanced prostate cancer (Fig 1). IRONMAN is a prospective, international registry recruiting a minimum of 5,000 patients with advanced prostate cancer (Table 1 and Fig 2) from 16 countries—the United States, Australia, Bahamas, Barbados, Brazil, Canada, Ireland, Jamaica, Kenya, Nigeria, Norway, South Africa, Spain, Sweden, Switzerland, and the United Kingdom (Fig 3). These initial 16 countries were selected on the basis of existing partnerships and the need to enhance participation in clinical studies by people of African ancestry.^16,17^ Within each country, we have sought to enroll patients from representative clinical centers to enroll individuals from diverse backgrounds. Patients will be followed for at least 5 years. The longer-term goal will be to expand the countries included in IRONMAN through new partnerships and additional funding. The overarching principle is to make this a lasting resource for the cancer research and clinical communities.

**TABLE 1:**
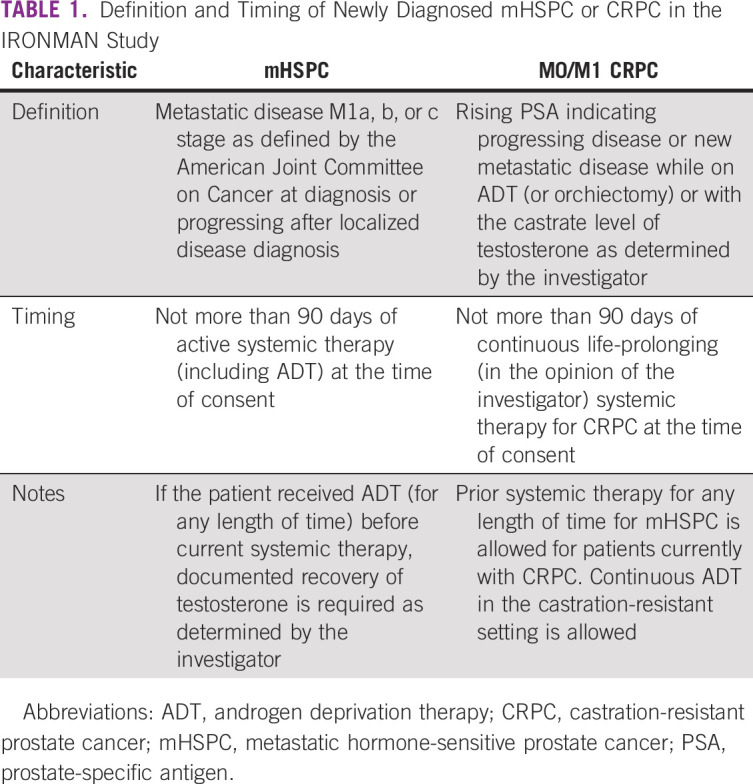
Definition and Timing of Newly Diagnosed mHSPC or CRPC in the IRONMAN Study

## PATIENTS AND METHODS

### Governance and Working Groups

**FIG 4:**
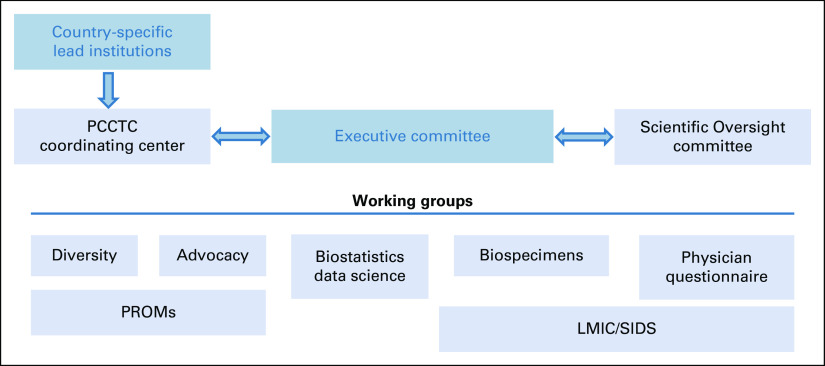
Scientific oversight and governance of the IRONMAN registry. LMIC, low- and middle-income countries; PCCTC, Prostate Cancer Clinical Trials Consortium; PROMs, patient-reported outcome measures; SIDSs, small island developing states.

IRONMAN has a governance structure to facilitate the study conduct and use of its resulting data. IRONMAN features working groups that work synergistically with the Executive Committee, the Clinical and Data Coordinating Center (The Prostate Cancer Clinical Trials Consortium, PCCTC), and the Scientific Advisory Committee (country lead investigators, industry funders as nonvoting members, and the advocacy community; Fig 4). The Executive Committee oversees all aspects of IRONMAN and is composed of the Principal Investigators, the PCCTC Chief Executive Officer, and the Executive Director of Programs at the Movember Foundation. The Executive Committee meets biweekly to review milestones, research proposals, and incorporate scientific advancements throughout the study.

Working groups are composed of investigators, study staff, patient advocates, and other scientists and clinicians within the IRONMAN community. The Diversity Working Group meets monthly to discuss barriers and strategies to enhance enrollment of a racially and ethnically diverse population.^17^ The Advocacy Working Group provides the patient perspective in all areas of IRONMAN. The Lower- and Middle-Income Countries (LMICs) and Small Island Developing States (SIDSs) Working Group was established to address the unique needs of patients recruited in the Caribbean and Africa and more broadly address oncology needs in these countries. The Biostatistics/Data Science Working Group brings statistical expertise, playing an essential role in assessing data quality and implementing analytic plans from investigators. The Biospecimen Working Group ensures high-quality biospecimen and biomarker collections and evaluates proposals for specimen use. Finally, the Patient-Reported Outcome Measures (PROMs) Working Group and the Physician Questionnaire Working Group monitor compliance of survey collection and address the respective data domains.

The PCCTC, a leader in the coordination of multicenter prostate cancer studies, is the global clinical and data coordinating center for IRONMAN. IRONMAN leverages the PCCTC's partnerships and infrastructure to manage the registry successfully and efficiently. Each participating country has a designated lead investigator and coordinator, whose institution serves as the local sponsor and coordinating center for their respective country. The PCCTC works closely with country leads to maintain regulatory oversight, patient privacy, and data quality.

### Under-Represented Populations

An unmet challenge in prostate cancer is the well-known racial disparity among people of African ancestry.^18,19^ In the United States, Black patients are more likely to present with advanced-stage prostate cancer^20-23^ and are 2.1 times more likely to die of prostate cancer compared with White patients.^24^ A similar disparity exists in the Caribbean. For example, Barbados has the second highest prostate cancer mortality rates in the world. However, several recently FDA-approved prostate cancer therapies are unavailable or inaccessible in LMICs and SIDSs.

Despite racial differences, clinical trials have generally had low enrollment of Black patients. In an analysis of 72 prostate cancer trials,^16^ 96% of patients were White. There is also a documented under-representation in clinical trials for patients living in rural areas despite higher mortality.^25,26^ Similarly, disparities in prostate cancer outcomes exist among US veterans, where cumulative prostate cancer–specific mortality is higher in patients treated in Veterans Affairs (VA) Medical Centers compared with the general population.^27^ IRONMAN aims to bridge gaps in knowledge through enrollment of a diverse population guided by the Diversity, LMIC/SIDS, and Advocacy Working Groups^17^ and through a global lens to enable equal benefit for all.

### Participating Countries and Sites

As of July 2022, IRONMAN is open and enrolling in 12 of 16 planned countries at 109 sites, with 14 additional sites planned for activation. US sites include NCI-designated cancer centers, community oncology practices, large group urology practices, and VA Medical Centers. Sites were selected to create breadth in the cohort across race/ethnicity, rural and urban populations, socioeconomic backgrounds, and geographic regions and include a sizable number of veterans. Each country has a diverse clinical setting on the basis of mixed local practices.

### Informed Consent and Eligibilty

**FIG 5:**
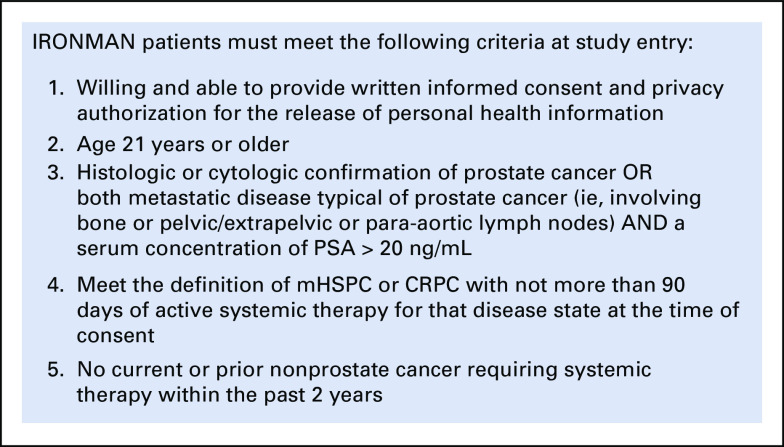
Eligibility criteria. CRPC, castration-resistant prostate cancer; mHSPC, metastatic hormone-sensitive prostate cancer; PSA, prostate-specific antigen.

The informed consent, protocol, and patient-facing materials are approved by local regulatory/ethics committees before site accrual. Patients who meet the eligibility criteria (Fig 5) are invited to enroll. The informed consent form allows collection of demographic and health information, future contacts, release of medical records including clinically significant adverse events, questionnaires, and collection of blood and genetic sequencing results. Patients are given opt-in opportunities for future research via access to their existing tissue and imaging assessments. The informed consent form and questionnaires have been translated into 14 languages and culturally adapted to the local customs of participating sites.

### Data Domains and Collection Methods

Guidelines from the International Consortium for Health Outcomes Measurement Advanced Prostate Cancer Working Group^28^ and Prostate Cancer Working Group 3^29^ were integrated to optimize data collection methods and assessments. Figure 2 outlines the timing and types of data collected. Data are gleaned from medical records, pathology reports, questionnaires, blood biospecimens, and physician questionnaires. IRONMAN leverages regular clinical visits of patients with advanced prostate cancer, which allows standardized assessments via participating sites.

Clinical data are captured via an Electronic Data Capture web-based platform. The PCCTC conducts automated edit checks, manual queries, and audit trails to ensure accurate, complete data collection. Demographics, PROMs, and patient-reported experience measures (PREMs) are captured via the electronic TrueNTH platform. On study registration, site staff enter the participant's e-mail address for those who consent to use TrueNTH. TrueNTH then prompts patients to complete questionnaires by automatic e-mail reminders where patients can access the questionnaires as they come due. Some patients and sites have elected to use paper questionnaires. All study data are transmitted via secure Internet connection from participating sites to a secure central database using encryption modalities.

### Biospecimens

Baseline and longitudinal blood specimens (Fig 2 and Table 2) are collected and then processed, aliquoted, and batch shipped to the global biorepository based at the Dana-Farber/Harvard Cancer Center Population Sciences Laboratory Support Core. Each ex-US country has a local biorepository where specimens are collected and stored before shipment to the United States. Baseline blood specimens are drawn after informed consent and not later than 90 days after beginning therapy. Follow-up collections are taken at the time of first change in treatment or at month 12, whichever occurs first. In addition, blood specimens are collected at each subsequent change in treatment because of progression of disease. A detailed laboratory manual with instructions on sample collection, processing, storage, and shipping has been developed. The unique patient identification number, date and time of blood draw, and originating tube are recorded for each blood draw. DNA extractions are periodically undertaken to assess DNA integrity for future biomarker studies. Use of biospecimens is governed by the Biospecimen Working Group and Executive Committee.

**TABLE 2:**
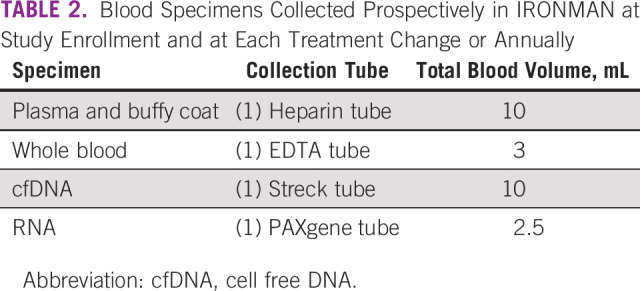
Blood Specimens Collected Prospectively in IRONMAN at Study Enrollment and at Each Treatment Change or Annually

### Patient-Reported Information

Demographic and epidemiologic data are collected at baseline including the following: height, weight (current and at age 40 years), smoking and other tobacco use, alcohol, marital status, living arrangement, education, employment status, physical activity, walking pace, sedentary behavior, use of multivitamins and supplements, race/ethnicity, military service, and family history of cancer (prostate, ovarian, breast, colon, melanoma, and pancreas) including family relation and age at cancer diagnosis.

PROMs and PREMs are collected at baseline and every three months (urinary function, sexual function, and PREMs are annual). On the basis of the International Consortium for Health Outcomes Measurement guidelines,^28^ IRONMAN uses the following validated instruments: EORTC QLQ-C30 v3, Brief Pain Inventory (severity, impact, and amount of pain), FACT-FPSI 17 (Functional Assessment of Cancer Therapy in patients with advanced prostate cancer), and EPIC-26 (Expanded Prostate Cancer Index Composite Short Form, urinary and sexual function questions). In addition, patients complete the Pittsburgh Sleep Quality Index on sleep quality, quantity, disruptions, and medications. Subjective cognitive function is assessed with questions derived from the Structured Telephone Interview for Dementia Assessment, which has been robustly validated^30,31^ and can identify patients in subclinical stages of cognitive impairment.^32-34^ Androgen deprivation therapy may impair cognitive function^35^ although the impact of newer androgen receptor–targeted therapies is unclear. Finally, six questions from the Cancer Australia's Core Patient Experience Indicators are used to collect data on PREMs. At some US sites and in Nigeria, Kenya, Jamaica, Barbados, and the Bahamas, the CaPTC-AC3 Behavioral and Epidemiological^36^ measures collect culturally responsive data from Black patients.

### Physician Questionnaire

There is a paucity of empirical data evaluating the reasons for treatment selection and discontinuation across oncology in real-world settings. To address this knowledge gap, we developed a novel questionnaire instrument. After creation of content domains, concepts were elicited through scripted interviews with urologists and medical oncologists in academic and community practices from seven countries with IRONMAN sites, and the draft questionnaire was pilot-tested. The final, English-language questionnaire is completed by the treating physicians at participant enrollment and with each subsequent discontinuation and initiation of a treatment. Domains covered include disease progression, toxicity, and reasons contributing to discontinuation of the prior treatment, as well as patient and tumor characteristics, comorbidities, patient preferences, and nonmedical reasons influencing treatment selection.

#### Prostate cancer treatments.

Prostate cancer treatments are documented at enrollment and longitudinally in the clinical database. Data include drug type, dose, dates of initiation and completion, and reason for treatment discontinuation. Treatments include newer agents (Appendix Table A1), older line therapies, and experimental agents.

#### Concomitant medications.

Concomitant medications are collected at baseline and every 12 months. Medications include aspirin, nonsteroidal anti-inflammatory drugs, statins, blood pressure lowering medications, diabetes medications, and antidepressants. Baseline information on supplements and multivitamins is collected via TrueNTH.

## RESULTS

### Clinical, Pathologic, and Outcome Data

At enrollment, sites provide pathologic and/or clinical stage, Gleason score, dates of prior treatment (prostatectomy, radiation, and hormonal therapy), and prostate-specific antigen level at diagnosis. The ECOG Scale of Performance Status is reported at enrollment and annually thereafter. Progression dates, location of metastases, and prostate-specific antigen are collected throughout the study period. Clinically significant adverse events, ie, serious adverse events and symptomatic skeletal events, including symptomatic pathologic fractures, palliative radiation therapy, palliative surgery, spinal cord compression, and other cancers, are also collected. Mortality (date and cause of death) is ascertained via report from sites, physician questionnaires, or searches of national database.

### Withdrawal From Ironman

Patients can withdraw participation in IRONMAN at any time, for any reason, and without consequence. If patients wish to discontinue, they are asked whether investigators may continue to collect data elements from their medical records including overall survival. Data collected before the patient's withdrawal of consent remain in the database, unless restricted by local regulations. Site staff indicate the date and reason for withdrawal in the database.

### Substudies

IRONMAN investigators, collaborators, and industry funders may develop concepts for independent or collaborative substudies leveraging the IRONMAN population and infrastructure. The proposed project must first be scoped by the PCCTC. Depending on its nature, the substudy proposal may be circulated to country lead investigators or applicable working groups for review and comment. If the substudy is determined to be feasible and in accordance with the overall goals and objectives of IRONMAN, the Executive Committee will approve the substudy proposal.

### Statistical Considerations

Although the study sample size was not powered for any one specific hypothesis, we seek to enroll sufficient numbers of patients to provide robust estimates in comparing outcomes separately for patients with mHSPC and CRPC, within a country, within racial/ethnic groups, and for clinical and molecular subtypes. The IRONMAN Biostatistics and Data Science working group has been an essential partner in determining the overall sample size of 5,000 patients across the registry. In addition, they have been integral in developing and executing biostatistical analysis plans for each of the study objectives and substudies.

### Progress to Date

**FIG 6:**
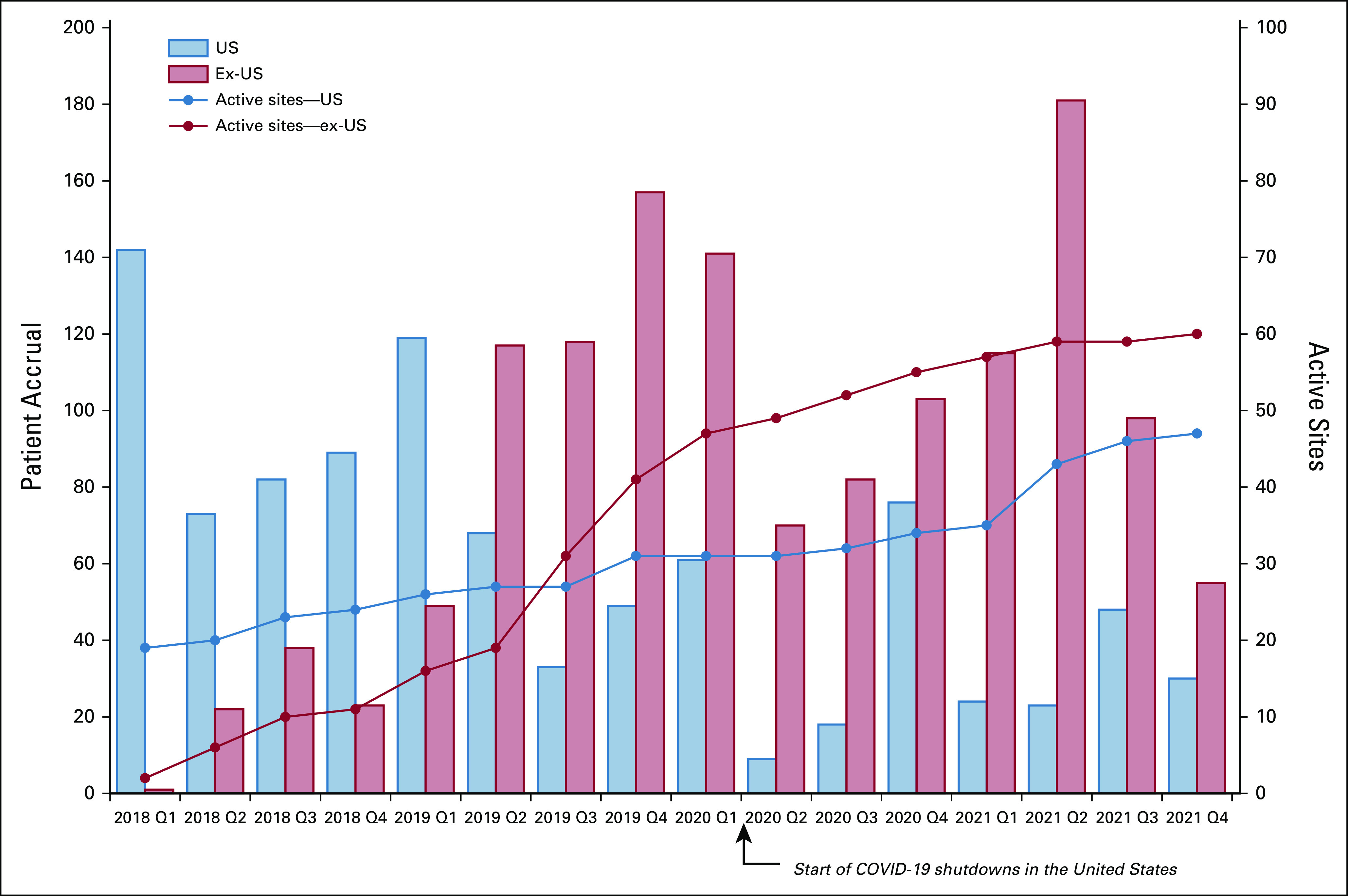
Site activations and quarterly accruals—US and ex-US sites since study inception.

To date, partnerships have been established and country lead investigators have been identified in all countries; sites in 12 of the 16 countries are open and enrolling. As of July 2022, 2,682 patients have been successfully enrolled—of whom 1,006 were enrolled from US sites. Figure 6 presents site activation and quarterly recruitment totals since study inception. Only 6% of patients have voluntarily withdrawn their participation from the study.

Table 3 presents baseline characteristics of the 2,682 eligible patients whose demographics are available as of July 2022. At the time of enrollment, 66% of patients had mHSPC and 34% had CRPC. The median (IQR) age at entry is 70 (64-76) years. On the basis of self-report, 11% of patients are Black and 9% are Hispanic. Five VA Medical Centers in the United States are activated for enrollment to date, and globally, 23% of patients report having served in the military.

**TABLE 3:**
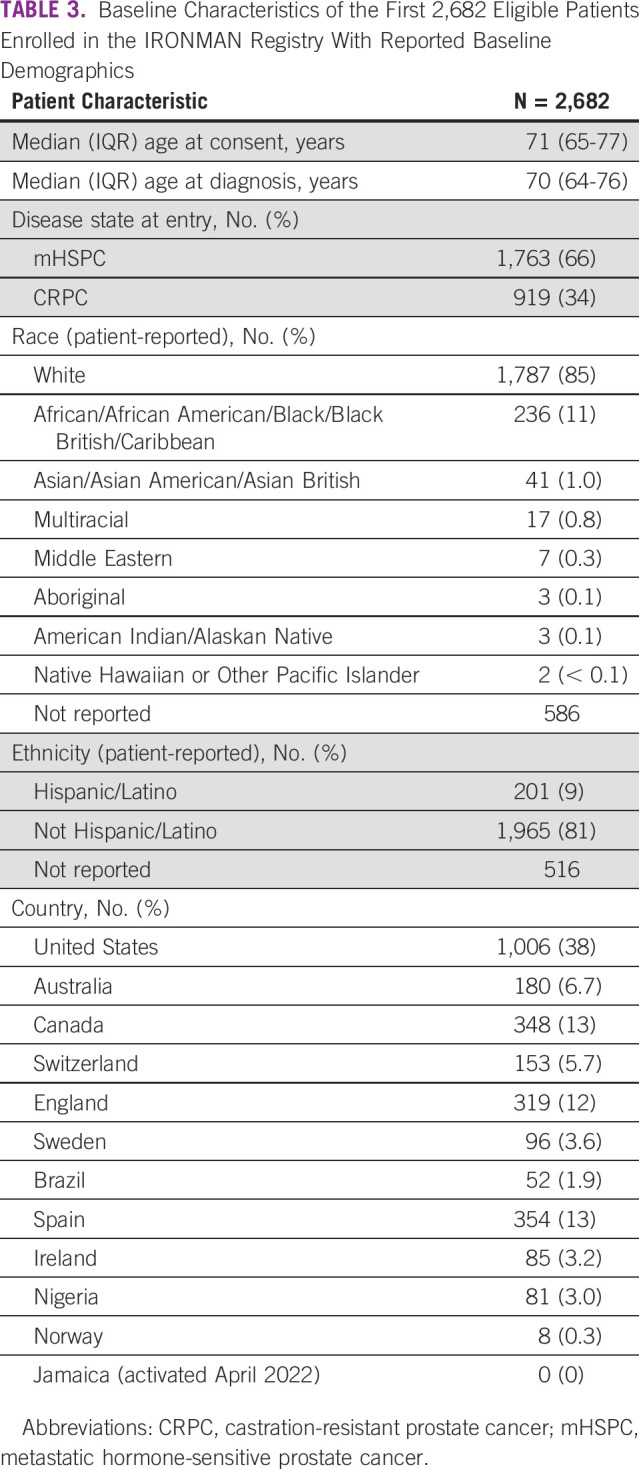
Baseline Characteristics of the First 2,682 Eligible Patients Enrolled in the IRONMAN Registry With Reported Baseline Demographics

Two primary challenges have affected recruitment and study conduct to date—the European Union's (EU's) General Data Protection Regulation (GDPR) and the COVID-19 pandemic. GDPR, effective May 2018, contains requirements related to the processing of personal data of individuals who are located within the EU and European Economic Area (EEA). GDPR applies to any organization that is processing personal data of individuals inside the EU/EEA, regardless of where the organization is located. The collection of IRONMAN data from EU/EEA sites is under the purview of GDPR regulations. Although this delayed opening sites in Europe for almost 12 months, the PCCTC has since executed data access agreements containing EU model clauses and designated an EU representative to serve as a trusted third-party contact per GDPR requirements.

The COVID-19 pandemic has affected IRONMAN in several ways. At almost all US sites, recruitment stopped completely at the beginning of the pandemic. The global impact has been variable given differences in COVIDg-19 infection, timing, and severity as well as variability in public health policies.^17^ Although recruitment in some sites has begun to trend toward prepandemic levels, particularly internationally, recruitment in others remains on hold. The pandemic has delayed opening new sites, including the activation of some sites in the Caribbean and Africa. The PCCTC developed a set of best practices for conducting clinical trials during the pandemic, and the study teams adapted to ensure the safety of its participants, clinicians, and research staff. The COVID-19 pandemic has also disrupted global supply chains, affecting the ability of country-specific biorepositories to reliably obtain the necessary biospecimen collection supplies and materials. As a result, the PCCTC implemented a centralized solution using a US-based vendor to obtain and ship supplies to the country-specific biorepositories.

### Future Directions

Sixteen countries are currently participating in IRONMAN, and we will seek to expand into additional regions. Although IRONMAN has been successful in recruiting Black patients, we need to continue along this trajectory and enhance recruitment of patients of Hispanic ethnicity and Asian ancestry. Currently, we are not enrolling in Asia or Eastern Europe and have limited representation in South America. This is a limitation of the registry, and we are working to identify opportunities for additional funding and engaging new partners to expand IRONMAN and enroll patients from a broader set of countries. We are in the process of expanding diversity initiatives across each country by identifying specific aspects of diversity that are important (eg, socioeconomic, private *v* public practices, and geography) and plan to work with each country lead investigator to set benchmarks for success.

We aim to enhance data collection types and repositories and conduct additional substudies. For example, we have institutional review board approval in place to collect archival prostate tumor tissue and clinical imaging assessments. Additional funding is needed to establish and maintain the infrastructure for these repositories.

A key unmet need among our LMIC/SIDS partners is the lack of accessibility to advanced prostate cancer therapies. We intend to establish initiatives aimed at increasing therapeutic access to patients in IRONMAN through diverse partnerships with pharmaceutical companies, foundations, governmental agencies, patients, and other stakeholders.

## DISCUSSION

The number of people globally diagnosed with advanced prostate cancer is considerable and growing because of advances in earlier detection and treatment, leading to longer survival. The treatment landscape has changed rapidly and will continue to do so. Randomized prospective trials to test all potential combinations in practice patterns are not feasible, but real-world evidence, such as that generated by IRONMAN, can help to inform potential optimal sequences of agents and identify subgroups of patients who would benefit the most.

Advanced prostate cancer and side effects of treatments likely affect quality-of-life issues. Relatively little is currently known about the real-life quality-of-life experience of these patients, beyond the narrowly defined patient populations within trials submitted for regulatory approvals. IRONMAN can elucidate which populations experience quality-of-life issues related to their treatments or disease by recruiting a diverse population and maintaining high levels of PROMs/PREMs compliance.

Generating real-world evidence will require data collection to be timely, thorough, and accurate. Although the COVID-19 pandemic, introduction of GDPR, and global differences in clinical research standards have posed challenges for recruitment and data collection, IRONMAN's diversified governance structure is well-positioned to meet these challenges and succeed in creating a valuable resource for the prostate cancer community.

## Figures and Tables

**TABLE A1:**
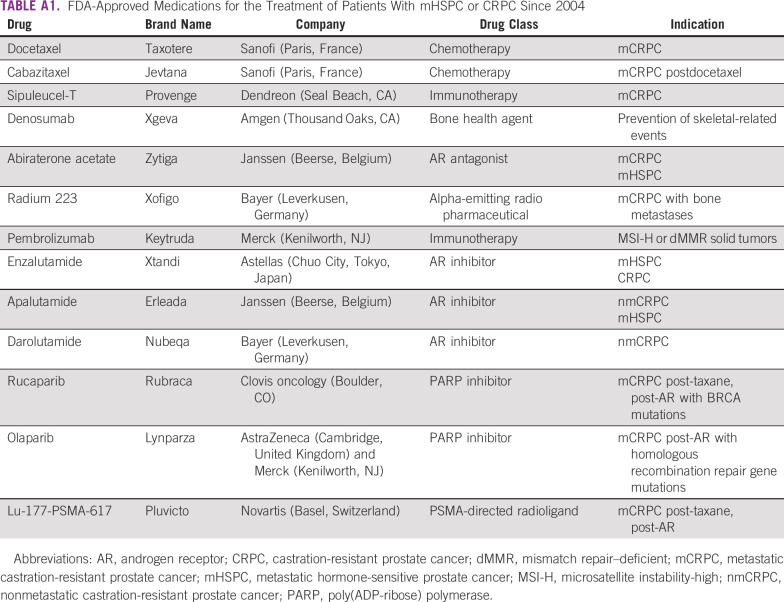
FDA-Approved Medications for the Treatment of Patients With mHSPC or CRPC Since 2004
